# Supplementary material for: FORWARD GENETICS IN C. ELEGANS REVEALS GENETIC ADAPTATIONS TO POLYUNSATURATED FATTY ACID DEFICIENCY
Source: bioRxiv. 2025 Jun 5:2024.11.08.622646. Preprint. [Version 3] doi: 10.1101/2024.11.08.622646 (PMC12157585; doi:10.1101/2024.11.08.622646)
Supplement: Supplement 1 [file nihpp2024.11.08.622646v3-supplement-1.pdf]

bioRxiv preprint doi: https://doi.org/10.1101/2024.11.08.622646; this version posted June 5, 2025. The copyright holder for this preprint (which was not certified by peer review) is the author/funder, who has granted bioRxiv a license to display the preprint in perpetuity. It is made available under aCC-BY 4.0 International license.

SUPPLEMENTARY FIGURES

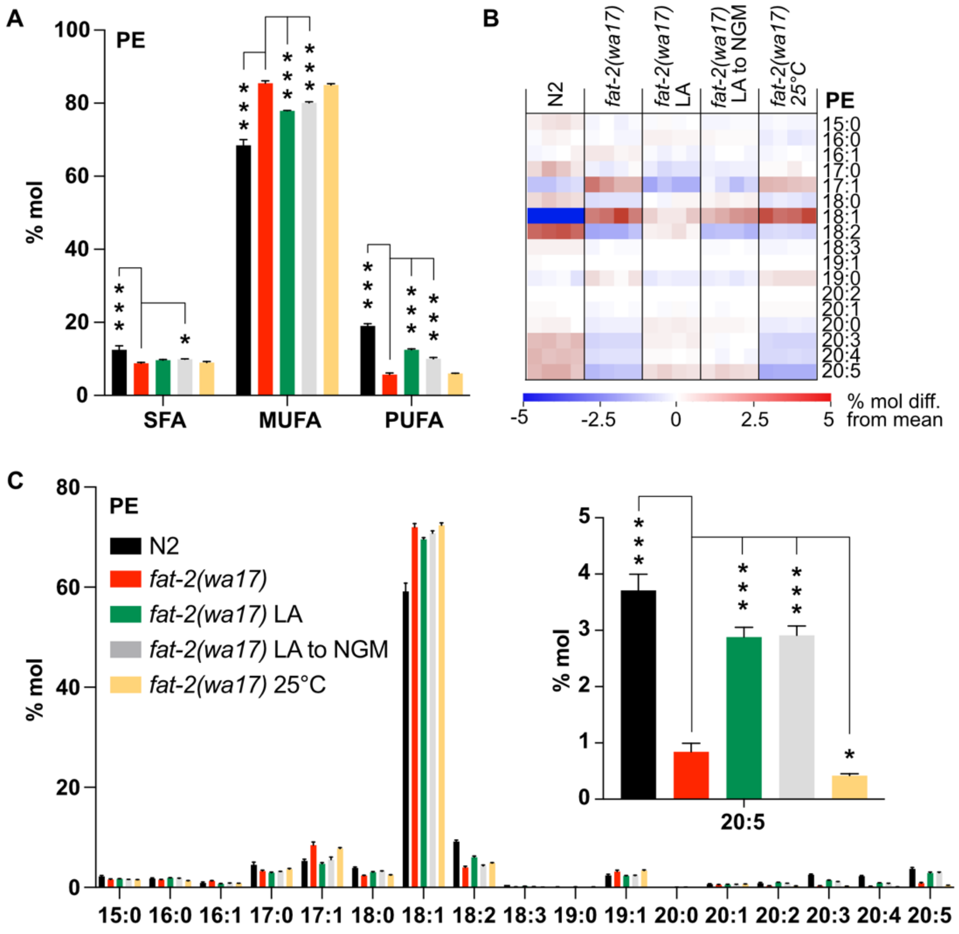

S1 Fig. Lipidomics analysis of PEs in *fat-2(wa17)* in various cultivation conditions. (A) SFA, MUFA, and PUFA levels in phosphatidylethanolamine (PEs) of *fat-2(wa17)* grown in various conditions. Cultivation on 2 mM LA boosts PUFA levels. LA to NGM worms were grown on 2 mM LA before being transferred to NGM 6 h prior to harvesting. **(B)** Heatmap of PE species in *fat-2(wa17)* in all conditions. **(C)** Levels of individual FA species in PEs for all conditions. Inset shows that the levels of C20:5 are increased by providing *fat-2(wa17)* with LA. *p < 0.05, **p < 0.01, ***p < 0.001 indicate significant differences compared to the *fat-2(wa17)* control.

bioRxiv preprint doi: https://doi.org/10.1101/2024.11.08.622646; this version posted June 5, 2025. The copyright holder for this preprint (which was not certified by peer review) is the author/funder, who has granted bioRxiv a license to display the preprint in perpetuity. It is made available under aCC-BY 4.0 International license.

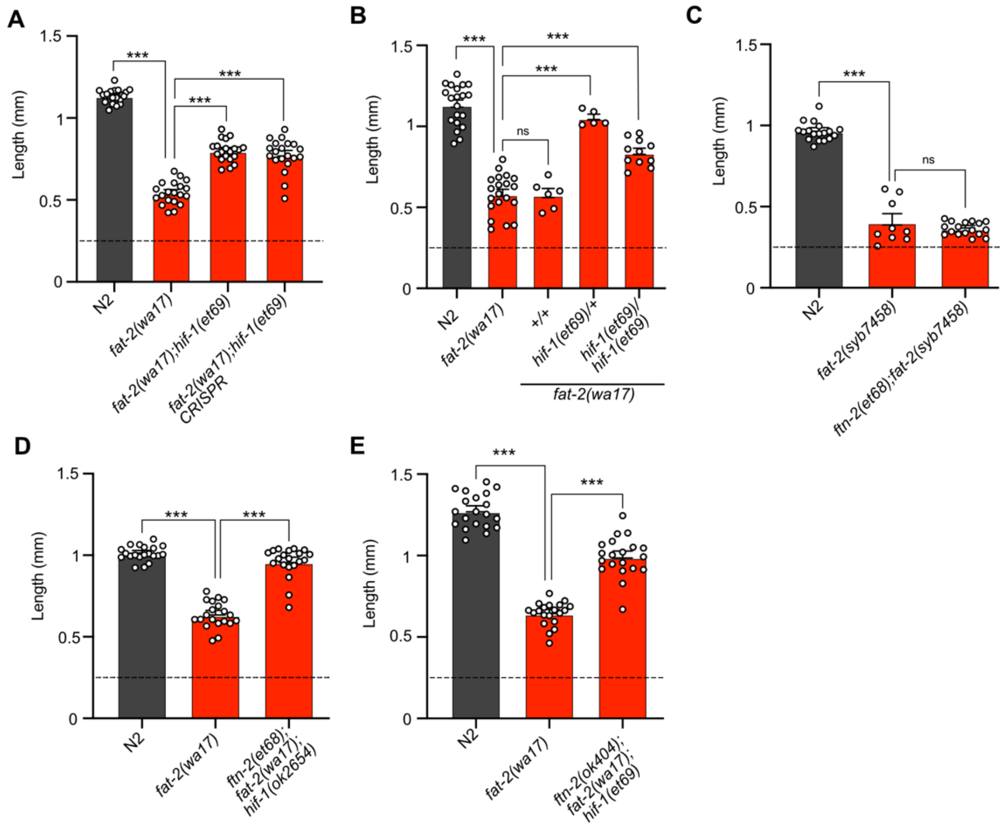

S2 Fig. *fat-2(wa17)* and *fat-2(syb7458)* with suppressors. (A) Confirmation of *hif-1(et69)* suppression of *fat-2(wa17)* by CRISPR-Cas9. **(B)** The *hif-1(et69)* allele acts best as a *fat-2(wa17)* suppressor when in a heterozygous state. **(C)** *ftn-2(et68)* does not act as a suppressor for the *fat-2(syb7458)* allele. **(D)** *ftn-2(et68)* still suppresses *fat-2(wa17)* in a *hif-1* null background. **(E)** *hif-1(et69)* suppresses *fat-2(wa17)* in a *ftn-2* null background. **(A-E)** All length measurements were taken 72 h after L1 synchronization. Horizontal dashed line represents the approximate size of L1s at the start of each experiment. Error bars show the standard error of the mean. *p < 0.05, **p < 0.01, ***p < 0.001 indicate significant differences compared to the *fat-2(wa17)* control.

bioRxiv preprint doi: https://doi.org/10.1101/2024.11.08.622646; this version posted June 5, 2025. The copyright holder for this preprint (which was not certified by peer review) is the author/funder, who has granted bioRxiv a license to display the preprint in perpetuity. It is made available under aCC-BY 4.0 International license.

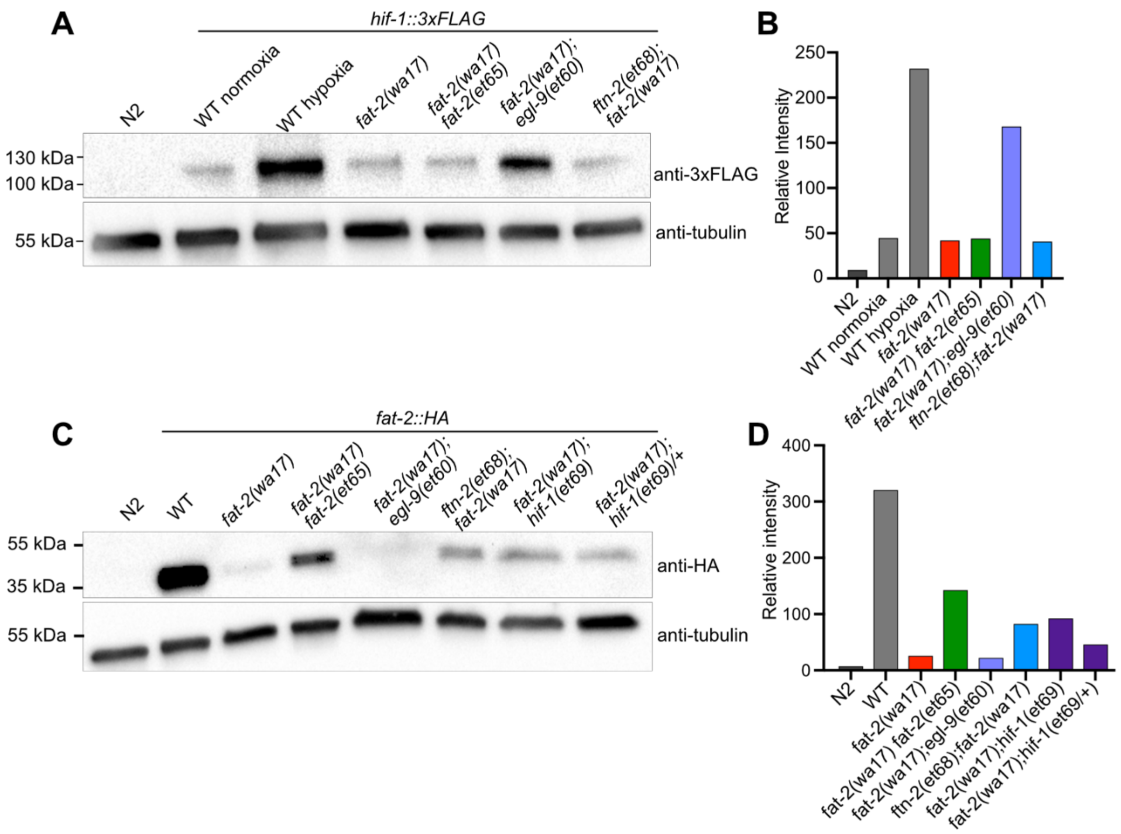

S3 Fig. Suppressors influence HIF-1 and FAT-2 levels. (A) Western blot showing that *hif-1::3xFLAG* levels in *fat-2(wa17)* are increased by *egl-9(et60)*. **(B)** Quantification of Western blot in A showing normalized relative intensity of the HIF-1 signal to that of tubulin. **(C)** Western blot showing that *fat-2::HA* levels are reduced in *fat-2(wa17)* but increased by suppressors. **(D)** Quantification of Western blot in C showing normalized relative intensity of HIF-1 signal to that of tubulin.

bioRxiv preprint doi: https://doi.org/10.1101/2024.11.08.622646; this version posted June 5, 2025. The copyright holder for this preprint (which was not certified by peer review) is the author/funder, who has granted bioRxiv a license to display the preprint in perpetuity. It is made available under aCC-BY 4.0 International license.

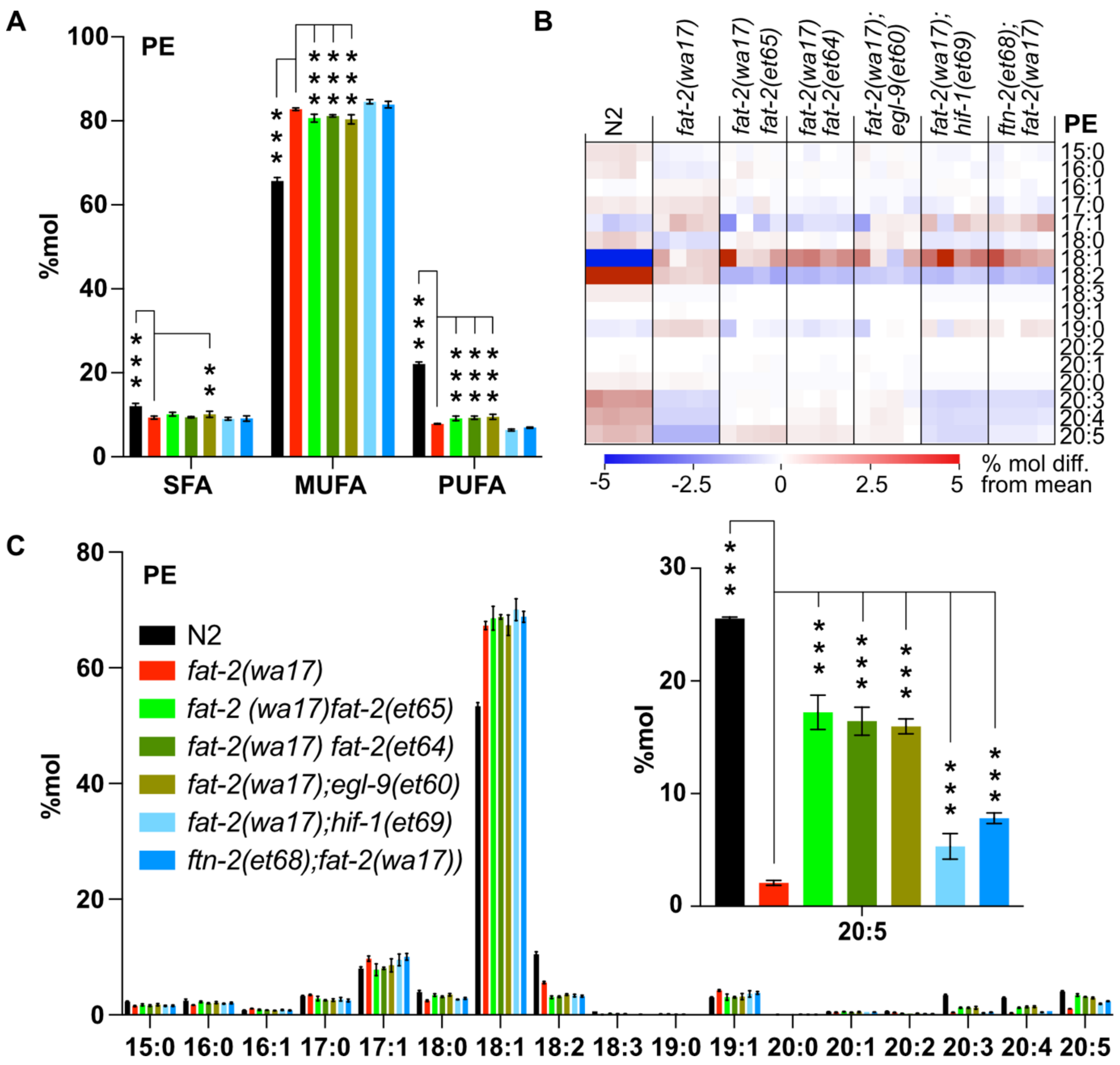

S4 Fig. Lipidomics of PEs in *fat-2(wa17)* suppressors. **(A)** Levels of SFAs, MUFAs, and PUFAs in PEs measured in *fat-2(wa17)* suppressors confirming that the suppressors increase PUFA levels in *fat-2(wa17)*. **(B)** Heat map analysis of PE species in suppressor mutants. **(C)** Levels of individual FA species in PEs in *fat-2(wa17)* suppressors, insert shows levels of 20:5 are increased in all double mutant strains. *p < 0.05, **p < 0.01, ***p < 0.001 indicate significant differences compared to the *fat-2(wa17)* control.

bioRxiv preprint doi: https://doi.org/10.1101/2024.11.08.622646; this version posted June 5, 2025. The copyright holder for this preprint (which was not certified by peer review) is the author/funder, who has granted bioRxiv a license to display the preprint in perpetuity. It is made available under aCC-BY 4.0 International license.

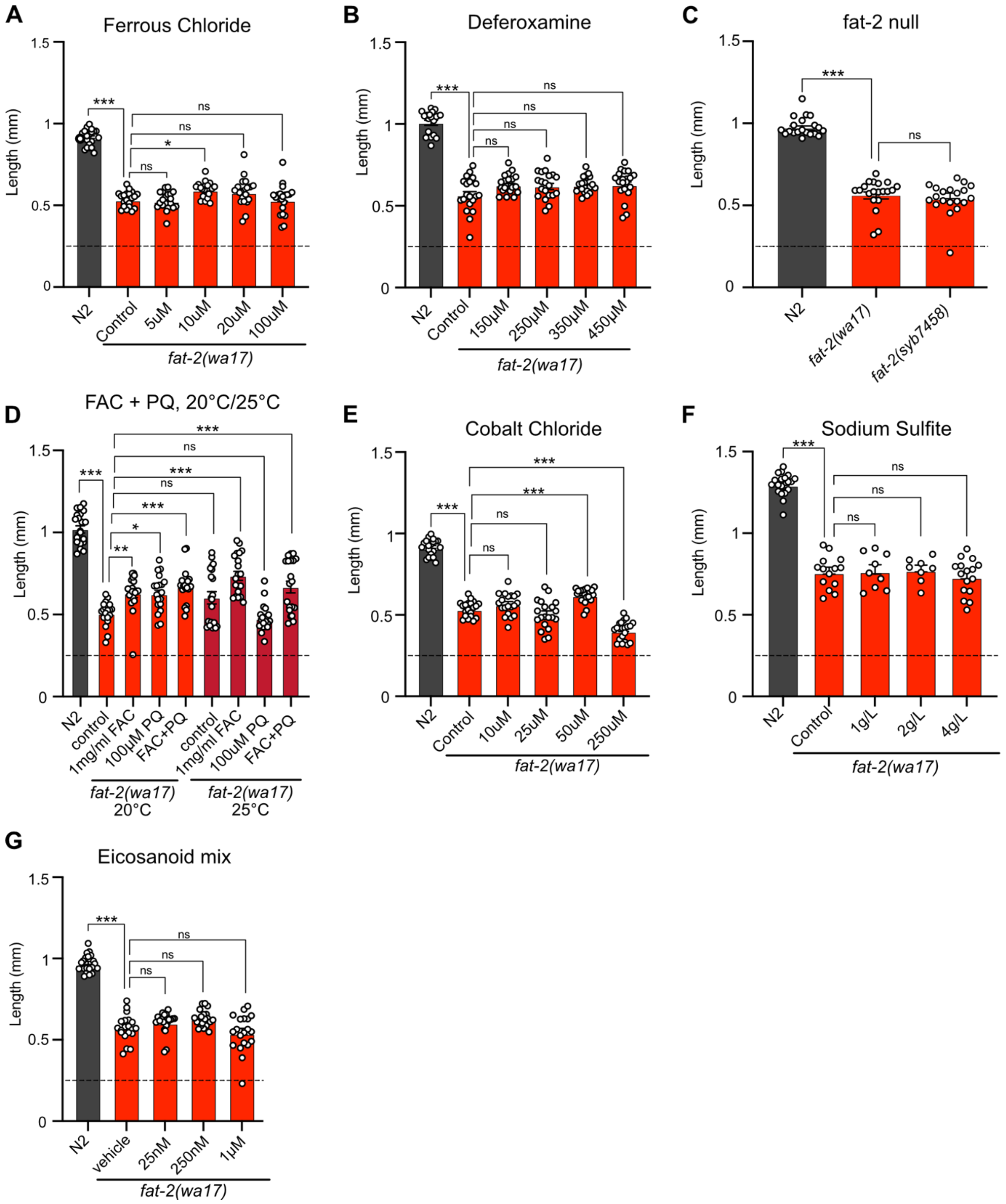

S5 Fig. Exogenous treatment of *fat-2(wa17)* to mimic suppressors. (A-E) Length of *fat-2(wa17)* treated with various diets for 72 h after L1 synchronization attempting to mimic effects of *egl-9*, *ftn-2*, and *hif-1* suppressors. Horizontal dashed line represents approximate size of worms at the start of each experiment. *p < 0.05, **p < 0.01, ***p < 0.001 indicate significant differences compared to the *fat-2(wa17)* control.

bioRxiv preprint doi: https://doi.org/10.1101/2024.11.08.622646; this version posted June 5, 2025. The copyright holder for this preprint (which was not certified by peer review) is the author/funder, who has granted bioRxiv a license to display the preprint in perpetuity. It is made available under aCC-BY 4.0 International license.

S1 Table. ***fat-2(wa17)*** **suppressor genotyping primers.**

Allele	Primer	Sequence	Expected product (bp)	Annealing temperature
fat-2(wa17)	Fwd	5' GACAATCGCTACAAAAGTG 3'	320	55°C
	WT Rev	5' CATTGAGCCATTCATAATTGG 3'		
	Mut Rev	5' CATTGAGCCATTCATAATTGA 3'		
fat-2(et63)	WT Fwd	5' AGGTACCGGAGCTTCCATTAG 3'	558	55°C
	Mut Fwd	5' AGGTACCGGAGCTTCCATTAA 3'		
	Rev	5' TGACACGATCCTCAGTAGTCTC 3'		
fat-2(et64-et66)	WT Fwd	5' CATGACTGTGGACATGGGTC 3'	334	55°C
	Mut Fwd	5' CATGACTGTGGACATGGGTT 3'		
	Rev	5' TGACACGATCCTCAGTAGTCTC 3'		
egl-9 (et60-et61)	WT Fwd	5' TGCCAGTCTTCCGTCGTCG 3'	739	55°C
	Mut Fwd	5' ATGCCAGTCTTCCGTCGTCA 3'		
	Rev	5' CGAACGACAAAACCGCGAAC 3'		
egl-9(et62)	WT Fwd	5' CATGCCAGTCTTCCGTCGTC 3'	741	57°C
	Mut Fwd	5' CATGCCAGTCTTCCGTCGTT 3'		
	Rev	5' CGAACGACAAAACCGCGAAC 3'		
ftn-2(et67)	WT Fwd	5' AGCCAGAGAATGATGAGCGG 3'	763	55°C
	Mut Fwd	5' AGCCAGAGAATGATGAGCGA 3'		
	Rev	5' AAGCCTTCAAGGCGGTTCCC 3'		
ftn-2(et68)	Fwd	5' ATGTCTCTCGCTCGTCAAAACT 3'	476	55°C
	WT Rev	5' AATAGAATAATCCTCACCTG 3'		
	Mut Rev	5' AATAGAATAATCCTCACCTA 3'		
hif-1(et69)	WT Fwd	5' GTGATTTCTTAACGTGTGTATTTTAG 3'	588	60°C
	Mut Fwd	5' GTGATTTCTTAACGTGTGTATTTTAA 3'		
	Rev	5' CATCATTGCTTCCGATGACTG 3'		
